# Dipeptidyl-peptidase IV inhibitor (DPP4i) confers increased odds of bullous pemphigoid even years after drug initiation

**DOI:** 10.1007/s00403-021-02317-9

**Published:** 2022-01-15

**Authors:** Khalaf Kridin, Orly Avni, Giovanni Damiani, Dana Tzur Bitan, Erez Onn, Orly Weinstein, Arnon D. Cohen

**Affiliations:** 1 Lübeck Institute of Experimental Dermatology, University of Lübeck, Ratzeburger Allee 160, 23562 Lübeck, Germany; 2 Azrieli Faculty of Medicine, Bar-Ilan University, Safed, Israel; 3 Unit of Dermatology and Skin Research Laboratory, Barch Padeh Medical Center, Poriya, Israel; 4 Clinical Dermatology, IRCCS Istituto Ortopedico Galeazzi, 20161 Milan, Italy; 5 Department of Biomedical, Surgical and Dental Sciences, University of Milan, 20122 Milan, Italy; 6 Department of Behavioral Sciences, Ariel University, Ariel, Israel; 7 Shalvata Mental Health Center, Affiliated with the Sackler School of Medicine, Tel Aviv University, Hod Hasharon, Israel; 8 Clalit Health Services, Tel-Aviv, Israel; 9 Faculty of Health Sciences, Ben-Gurion University of the Negev, Ben-Gurion Ave, Beer Sheva, Israel

**Keywords:** DPP4i, Gliptin, Bullous pemphigoid, BP

## Abstract

The timing pattern in which dipeptidyl-peptidase IV inhibitors (DPP4i) confer the risk of bullous pemphigoid (BP) is unknown. To investigate the odds of BP following exposure to DPP4i and to perform a duration-response analysis evaluating the risk of BP in relation to the duration of exposure to the culprit drug. A population-based nested case–control study was performed comparing diabetic patients with BP (*n* = 1458) with age-, sex- and ethnicity-matched diabetic control subjects (*n* = 6051) with respect to the prevalence of exposure to DPP4i. Adjusted odds ratios (ORs) were estimated by logistic regression. Overall exposure to DPP4i was associated with an 80% increase in the odds of subsequent BP (OR, 1.81; 95% CI, 1.46–2.08; *P* < 0.001). In an intraclass analysis, the odds of BP were increased in association with vildagliptin (OR, 3.40; 95% CI, 2.69–4.29; *P* < 0.001) and sitagliptin (OR, 1.56; 95% CI, 1.33–1.84; *P* < 0.001). In a duration-response analysis, the highest likelihood of BP was found 1–2 years after commencing the drug (OR, 2.66; 95% CI, 1.97–3.59; *P* < 0.001). The odds of BP were increased across all time periods and retained its statistical significance even ≥ 6 years after the drug initiation (OR, 1.44; 95% CI, 1.09–1.91; *P* = 0.011). Relative to other diabetic patients with BP, patients with DPP4i-associated BP were more likely to be admitted to inpatient dermatologic wards (OR, 1.66; 95% CI, 1.30–2.13; *P* < 0.001) and had higher mean(SD) numbers of outpatient dermatologist visits (14.7[14.8] vs. 12.3[13.2], respectively; *P* = 0.006). DPP4i should be suspected as a predisposing factor for BP even numerous years after the drug initiation.

**Supplementary Information:**

The online version contains supplementary material available at 10.1007/s00403-021-02317-9.

## Introduction

The last two decades witnessed a substantial increase in the incidence of bullous pemphigoid (BP) [1]. This epidemiological observation has been ascribed to multiple factors, including the increasing exposure to culprit medications [1]. Multiple lines of evidence denoted that dipeptidyl-peptidase IV inhibitors (DPP4i), or gliptins, second- to third-line oral antidiabetic drugs, are associated with an increased risk of developing BP [2–8].

While DPP4i emerged as an indisputable risk factor of BP [9], a debate is still surrounding the question of whether DPP4i-associated BP is typified by a unique morphological, immunological, genetic, or histologic profile distinguishing it from typical BP [5, 8, 10–18]. Another crucial unanswered question relates to the existence of duration-response relation between exposure to DPP4i and the emergence of BP. To elaborate, inconsistency still exists with regard to the time in which DPP4i confers the highest risk of BP development.

The aim of the current study is to evaluate the odds of BP under different DPP4i agents. A specific spotlight will be shed on a duration-response analysis evaluating the risk of BP in relation to the duration of exposure to the culprit drugs. The secondary endpoint is to delineate the clinical and prognostic outcomes of patients with DPP4i-associated BP as compared to other diabetic patients with BP.

## Methods

### Study design and dataset

The current study was designed as a population-based nested case–control study. The computerized database of Clalit Health Service (CHS) was the origin of the current study. The study was approved by the Institutional Review Board of CHS (0212-17-COM).

CHS is the largest health maintenance organization in Israel, ensuring 4.6 million enrollees, which represent 51% of the general Israeli population. The computerized database of CHS continuously retrieves data from all tiers of healthcare facilities, including primary healthcare services, outpatient referral clinics, and inpatient wards. The database undergoes constant logarithmic checks to ascertain the validity of the diagnoses. The loss to follow-up is minor and access to CHS services is free, thus rendering the database compatible with generating robust epidemiological data. Further characteristics of CHS database are detailed in our previous publications [19–21].

### Study population and definition of variables

The CHS database was systematically screened for incident cases with a diagnostic code BP and type 2 diabetes mellitus (T2DM) between the years 2007 and 2019. The diagnosis of BP was based on at least one of the following eligibility criteria: (i) a documented diagnosis of BP documented at least twice by a board-certified dermatologist, or (ii) a diagnosis of BP in discharge letters from dermatological wards. The diagnosis of T2DM relied on one of the following criteria: (i) two random tests of blood glucose greater than 200 mg/dL, (ii) one random test of blood glucose over 200 mg/dL with proven target organ damage, or (iii) two fasting glucose tests over 126 mg/dL.

A control group including 4–5 individuals per each case of BP was additionally enrolled. All control individuals lacked a diagnosis of BP but had a diagnosis of T2DM. Controls were matched based on sex, 5-year age group, and ethnicity and were recruited on the day in which the corresponding case was diagnosed.

Exposure to DPP4i agent was defined when sitagliptin, vildagliptin, linagliptin, or saxagliptin were prescribed for at least one month. Given uncertainties related to the timing of onset reported in the literature, the following durations of exposure to DPP4i have been arbitrarily chosen in the duration-response analysis (< 1, 1–2, 2–4, 4–6, ≥ 6 years). Outcome measures were adjusted for Charlson comorbidity index (CCI), an epidemiological scale that estimates the extent and severity of the comorbidities of each study participant. This index is widely utilized in epidemiological studies and was proved reliable in predicting mortality [22].

Since we did not have direct access to severity scores of patients, the burden of the disease was indirectly evaluated by the therapeutic regimen (e.g., the need for systemic corticosteroids and immunosuppressive/immunomodulatory adjuvant drugs) and healthcare utilization. Long-term systemic and topical corticosteroid variables were defined in cases managed by any drug pertaining to these classes for ≥ 6 months. Immunosuppressive/immunomodulatory adjuvant drugs included methotrexate, azathioprine, mycophenolate mofetil, rituximab, intravenous immunoglobulins, and plasmapheresis. Number of admission to dermatologic wards as well as number of visits to outpatient dermatologists following the diagnosis of BP was evaluated to provide an indirect estimate of disease severity.

### Statistical analysis

Baseline characteristics were described by means and standard deviations (SD)s for continuous variables, whilst categorical values were signified by percentages. The comparison between different subgroups was performed using the Chi-square test and *t*-test, as indicated.

Logistic regression was used to calculate odds ratios (ORs) and 95% confidence intervals (CI)s to compare cases and controls regarding the presence of preceding DPP4i exposure. Owing to the temporal relationship between exposure and outcome in case–control studies, the association was calculated only based on individuals who developed BP after DPP4i. Two-tailed *P*-values less than 0.05 were considered statistically significant. Differences in the all-cause mortality of DPP4i-associated BP and diabetic patients with non- DPP4i-associated BP were evaluated using a stratified log-rank test. All statistical analyses were performed using SPSS software, version 25 (SPSS, Armonk, NY: IBM Corp).

## Results

### Characteristics of the study participants

The current study population comprised 7509 participants, of whom 1458 were diabetic patients with BP and 6051 were diabetic control subjects. The mean (SD) age at the diagnosis of diabetic patients with BP was 76.9 (11.0) years, 676 (46.4%) were males, and 1380 (94.7%) were of Jewish ancestry (Table 1). Patients with BP experienced an increased burden of comorbidities relative to controls, as demonstrated by a higher mean (SD) CCI score (3.4 [2.4] vs. 3.0 [2.3], respectively; *P* < 0.001). Demographic and clinical features of the study participants are detailed in Table 1.

**Table 1:** Descriptive characteristics of the study population

Characteristic	Diabetic patients with BP (*N* = 1458)	Diabetic controls (*N* = 6051)	*P* value
Age, years			
Mean (SD)	76.9 (11.0)	77.7 (10.4)	**0.011**
Median (range)	78.6 (4.9–104.4)	79.4 (10.7–102.8)	
Sex, *N* (%)			
Male	676 (46.4%)	2731 (45.1%)	0.396
Female	782 (53.6%)	3320 (54.9%)	
Ethnicity, *N* (%)			
Jews	1,380 (94.7%)	5690 (94.0%)	0.368
Arabs	78 (5.3%)	361 (6.0%)	
BMI, mg/kg^2^			
Mean (SD)	29.1 (6.2)	28.8 (6.0)	0.103
Smoking, *N* (%)	512 (35.1%)	2105 (34.8%)	0.813
Charlson comorbidity score^a^			
Mean score (SD)	3.4 (2.4)	3.0 (2.3)	** < 0.001**

*BP* bullous pemphigoid; *N* Number; *SD* standard deviation; *BMI* body mass index

Significant values are in bold

^a^Without diabetes mellitus

### The odds of bullous pemphigoid following the exposure to DPP4i

Out of eligible patients with BP, 322 (22.1%) have been managed by DPP4i prior to the development of their disease. Sitagliptin was the most frequent agent (*n* = 234), followed by vildagliptin (*n* = 134), linagliptin (*n* = 12), and saxagliptin (*n* = 7), with 65 patients being managed by more than an individual DPP4i agent.

Overall exposure to DPP4i was associated with an 80% increase in the odds of subsequent BP (OR, 1.81; 95% CI, 1.46–2.08; *P* < 0.001). In an intraclass analysis, the odds of BP were highest in association with vildagliptin (OR, 3.40; 95% CI, 2.69–4.29; *P* < 0.001) and were additionally increased among those treated by sitagliptin (OR, 1.56; 95% CI, 1.33–1.84; *P* < 0.001). Linagliptin (OR, 0.61; 95% CI, 0.33–1.13; *P* = 0.113) and saxagliptin (OR, 1.39; 95% CI, 0.59–3.27; *P* = 0.451) were not significantly associated with the development of BP (Table 2).

**Table 2:** The odds of bullous pemphigoid following exposure to different DPP4i agents

Disease	Prevalence in diabetic BP patients, *n* (%)	Prevalence in diabetic controls, *n* (%)	Unadjusted OR (95%CI) [*P* value]	Male-specific OR (95%CI) [*P* value]	Female-specific OR (95%CI) [*P* value]	≥ 78.6 years-specific OR (95%CI) [*P* value]	< 78.6 years-specific OR (95%CI) [*P* value]	Adjusted OR (95%CI)^a^ [*P* value]
Overall DPP4i^a,b^	322 (22.1%)	823 (13.6%)	**1.80 (1.56–2.08) [< 0.001]**	**1.97 (1.61–2.40) [< 0.001]**	**1.63 (1.32–2.01) [< 0.001]**	**1.68 (1.36–2.06) [< 0.001]**	**1.92 (1.57–2.34) [< 0.001]**	**1.86 (1.61–2.16) [< 0.001]**
Sitagliptin^a,b^	234 (17.3%)	642 (11.8%)	**1.56 (1.33–1.84) [< 0.001]**	**1.57 (1.25–1.98) [< 0.001]**	**1.55 (1.23–1.95) [< 0.001]**	**1.44 (1.14–1.82) [0.002]**	**1.67 (1.33–2.09) [< 0.001]**	**1.60 (1.36–1.88) [< 0.001]**
Vildagliptin^a,b^	134 (9.4%)	174 (2.9%)	**3.40 (2.69–4.29) [< 0.001]**	**3.69 (2.71–5.04) [< 0.001]**	**3.04 (2.13–4.33) [< 0.001]**	**2.97 (2.09–4.22) [< 0.001]**	**3.74 (2.73–5.12) [< 0.001]**	**3.47 (2.75–4.39) [< 0.001]**
Linagliptin^a,b^	12 (0.9%)	80 (1.4%)	0.61 (0.33–1.13) [0.113]	0.85 (0.41–1.76) [0.662]	0.33 (0.10–1.08) [0.054]	0.67 (0.30–1.50) [0.327]	0.55 (0.22–1.41) [0.209]	0.68 (0.26–1.75) [0.425]
Saxagliptin^a,b^	7 (0.5%)	21 (0.3%)	1.39 (0.59–3.27) [0.451]	1.63 (0.51–5.20) [0.408]	1.16 (0.32–4.17) [0.821]	1.60 (0.56–4.37) [0.385]	1.12 (0.23–5.42) [0.884]	1.35 (0.28–6.57) [0.714]

*BP* bullous pemphigoid; *N* Number; *OR* odds ratio; *CI* confidence interval. *DPP4i* Dipeptidyl peptidase‐4 inhibitor

Significant values are in bold

^a^Multivariate logistic regression model adjusting for age, sex, ethnicity, and comorbidities (per CCI)

^b^65 patients were managed by more than a single DPP4i agent

^c^Patients managed by these drugs after the onset of BP (in cases) and recruitment (in controls) were omitted from the analysis

In a sex-stratified analysis, the odds of BP were more prominent among males undergoing overall DPP4i (OR, 1.97; 95% CI, 1.61–2.40; *P* < 0.001) and vildagliptin (OR, 3.69; 95% CI, 2.71–5.04; *P* < 0.001) treatment. When patients were divided on the basis of the median age, those younger than 78.6 years demonstrated a higher likelihood of BP following overall DPP4i (OR, 1.92; 95% CI, 1.57–2.34; *P* < 0.001), vildagliptin (OR, 3.74; 95% CI, 2.73–5.12; *P* < 0.001), and sitagliptin (OR, 1.67; 95% CI, 1.33–2.09; *P* < 0.001; Table 2). The outcome measures have not altered meaningfully in a multivariate analysis adjusting for age, sex, ethnicity, and comorbidities (Table 2).

### A duration-response analysis estimating the odds of BP in relation to the duration of exposure

**Fig. 1:**
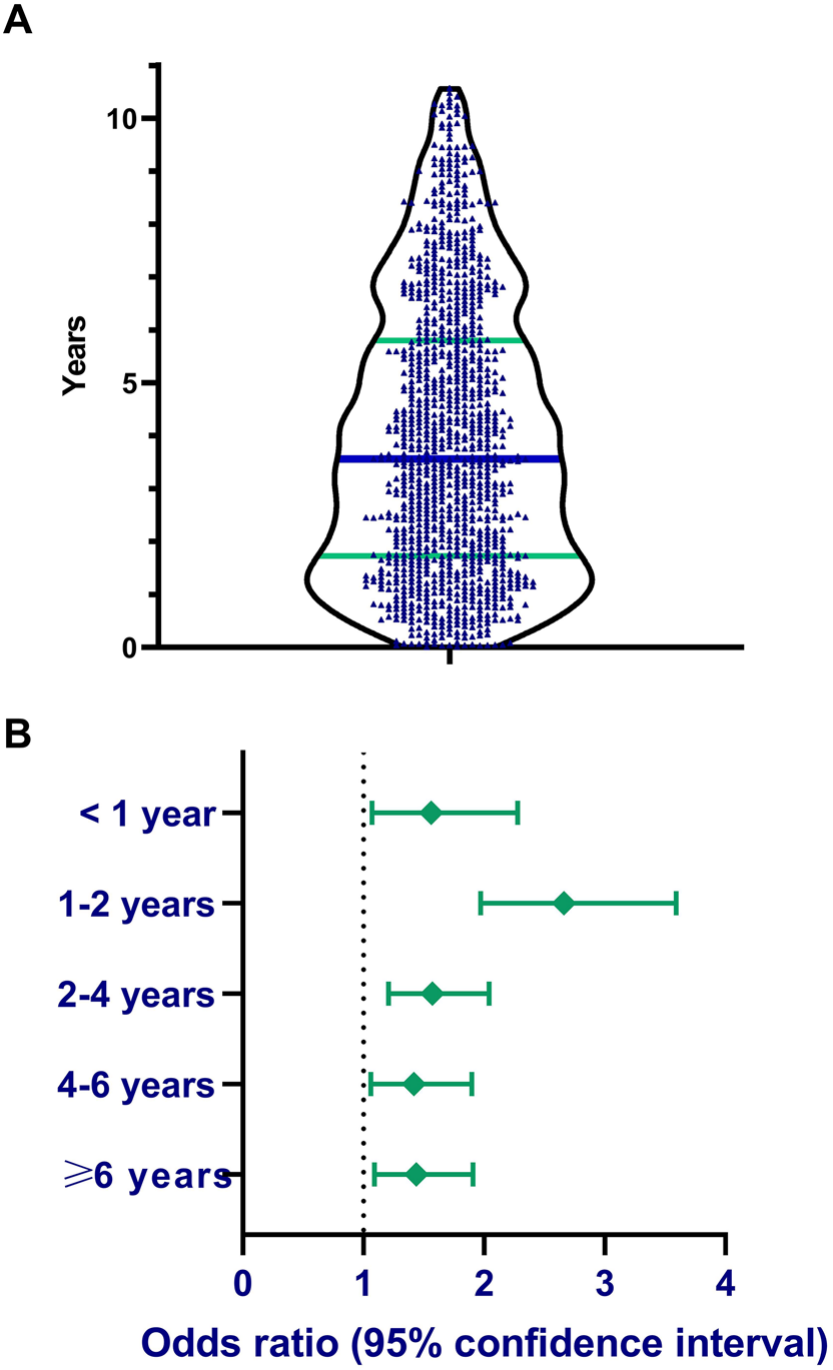
**A** A violin plot demonstrating distribution of latency between DPP4i initiation and onset of bullous pemphigoid. The blue bar represents the median and the green bars represent 25th and 75th quartiles. **B** Odds ratio of BP in different durations of exposure to DPP4i

The median (range) latency separating the start of exposure to DPP4i agents and the development of BP was 3.3 (0.1–10.3) years (Fig. 1A). When the odds of DPP4i-associated BP were evaluated in relation to the duration of exposure, the highest likelihood of BP was found 1–2 years after commencing the drug (OR, 2.66; 95% CI, 1.97–3.59; *P* < 0.001). The risk was increased across all time periods and retained its statistical significance even ≥ 6 years following the drug initiation (OR, 1.44; 95% CI, 1.09–1.91; *P* = 0.011; Fig. 1B).

### Clinical characteristics of patients with DPP4i-associated BP relative to other diabetic patients with BP

Table 3 delineates the clinical features of patients with DPP4i-associated BP (*n* = 322) as compared to diabetic patients with non-DPP4i-associated BP (*n* = 1136). Patients in the former group were typified by a male preponderance (OR, 1.66; 95% CI, 1.30–2.13; *P* < 0.001) and a higher frequency of smoking (OR, 1.36; 95% CI, 1.05–1.75; *P* = 0.018). With regard to the therapeutic approach, patients with DPP4i-associated BP were more likely to be admitted to inpatient dermatologic wards (OR, 1.66; 95% CI, 1.30–2.13; *P* < 0.001) and had higher mean (SD) numbers of outpatient dermatologists visits (14.7 [14.8] vs. 12.3 [13.2], respectively; *P* = 0.006) after their diagnosis.

**Table 3:** The clinical characteristics of patients with DPP4i-associated BP relative to their diabetic non-DPP4i-associated BP counterparts

	DPP4i-associated BP (*n* = 322)	Diabetic non-DPP4i-associated BP (*n* = 1136)	OR (95% CI)	*P* value
Age at the onset of BP, years; mean (SD)	77.3 (8.3)	76.8 (11.6)	1.04 (0.93–1.17)^a^	0.477
Male sex, *n* (%)	181 (56.2%)	495 (43.6%)	**1.66 (1.30–2.13)**	** < 0.001**
Jewish ethnicity, *n* (%)	306 (95.0%)	1,074 (94.5%)	1.10 (0.63–1.94)	0.731
Smoking, *n* (%)	131 (40.7%)	381 (33.5%)	**1.36 (1.05–1.75)**	**0.018**
Charlson Comorbidity Score; mean (SD)	3.2 (2.3)	3.5 (2.4)	0.95 (0.90–1.00)	0.062
Long-term systemic corticosteroids, *n* (%)^b^	224 (69.6%)	769 (67.7%)	1.09 (0.83–1.43)	0.525
Long-term topical corticosteroids, *n* (%)^c^	313 (97.2%)	1,080 (95.1%)	1.80 (0.88–3.69)	0.101
Adjuvant immunosuppressive or immunomodulatory agents^d^, *n* (%)	18 (5.6%)	72 (6.3%)	0.88 (0.51–1.49)	0.623
Admission to inpatient dermatologic wards, *n* (%)	95 (29.5%)	263 (23.2%)	**1.39 (1.05–1.83)**	**0.019**
Length of stay in inpatient dermatologic wards, days; mean (SD)	5.6 (11.6)	4.3 (10.9)	1.01 (1.00–1.02)^**e**^	0.057
Number of visits to outpatient dermatologists; mean (SD)	14.7 (14.8)	12.3 (13.2)	**1.01 (1.01–1.02)**^**f**^	**0.006**

*BP* bullous pemphigoid; number; *SD* standard deviation; *DPP4i* Dipeptidyl peptidase‐4 inhibitor

Significant values are in bold

^a^OR per 10-year increase in age

^b^Patients managed by systemic corticosteroids for more than 6 months

^c^Patients managed by topical corticosteroids for more than 6 months

^d^ Patients managed by one of the following agents: azathioprine, mycophenolate mofetil, methotrexate, cyclophosphamide, rituximab, plasmapheresis, intravenous immunoglobulins

^e^OR per day of hospital stay in age

^f^ OR per visit

We then compared the risk of all-cause mortality among the two aforementioned subgroups. After adjusting for age, sex, ethnicity, and comorbidities, the risk of all-cause mortality was comparable between patients with DPP4i-associated BP and diabetic patients with non- DPP4i-associated BP (HR, 0.85; 95% CI, 0.68–1.05; *P* = 0.125; Supplementary Fig. 1).

## Discussion

The current population-based study depicted that exposure to DPP4i was associated with an 80% increase in the likelihood of subsequent BP. The odds of BP peaked 1–2 years following the initiation of DPP4i and remained statistically significant for more than 6 years after drug initiation. Compared with other diabetic patients with BP, those with DPP4i-associated BP displayed a greater frequency of admissions to inpatient dermatologic wards and visits to outpatient dermatologists.

The knowledge about the risk of BP with DPP4i therapy stemmed originally from anecdotal case reports and national pharmacovigilance database analyses [23, 24], and was subsequently authenticated by numerous observational controlled studies [2–8]. A recent meta-analysis has revealed that exposure to DPP4i was associated with more than a threefold increased risk of developing BP (pooled OR, 3.16; 95% CI 2.57–3.89) [9].

The current study revealed an 80% increased odds of BP under DPP4i. This estimate is lower than the pooled OR of the quantitative synthesis [9] and particularly than a large-scale case–control Finnish study reporting an OR of 3.45 (95% CI, 2.69–4.44) [3]. Since the latter study did not set a history of T2DM as an eligibility criterion for cases and controls, it was very likely to overestimate the association given that patients with BP are at an increased risk of T2DM at baseline. To elaborate further, the high OR probably mirrors the increased frequency of T2DM in BP rather than the predisposing effect of DPP4i [3]. Our estimate is closer to the hazard ratios (1.42 [95% CI, 1.17–1.72] [7] and 2.2 [95% CI, 1.45–3.38] [6]) provided by two large-scale retrospective cohort studies estimating the incidence of BP among patients with T2DM placed on DPP4i relative to second-line antidiabetic drugs. These two observational studies, typified by a robust design and large study population, were statistically and methodologically powered to investigate the association of BP with DPP4i and were not included in the aforementioned meta-analysis [9].

In congruence with the vast majority of other studies [2, 3, 5, 10], vildagliptin was implicated with the strongest potential of triggering BP. Vildagliptin is typified by a relatively lower selectivity for the DPP4 enzyme in comparison with other members of the DPP family, such as DPP-8 and DPP-9 [25]. Therefore, it has been assumed that off-target DPP-8/DPP-9 inhibition might account for the excessive risk associated with this individual drug [7]. In the current study, sitagliptin imposed a statistically significant risk of eliciting BP. While this finding aligns with the observation of Varpuluoma et al. [3], it negates other studies signifying that sitagliptin is not associated with BP [2, 5, 7, 10]. Although linagliptin was found to predispose diabetic patients to BP in several studies [2, 5–7], the current study failed to reproduce this observation, probably due to the few exposed events. Due to the low number of patients under saxagliptin, the current study, similar to three recent studies [6–8], was underpowered to gauge the odds of saxagliptin-associated BP.

The pattern of timing in which DPP4i leads to the development of BP is a question with enormous clinical implications. The median latency between the commencement of DPP4i and the onset of BP varied noticeably in different reports, ranging between 6.0 to 26.4 months [2, 3, 5, 8, 13, 23]. The median latency in our cohort (3.3 years) was higher than all previous reports. Temporal trends in the risk of BP throughout the duration of exposure to DPP4i were evaluated in a single study and were found to peak after 20 months of treatment [6]. This finding accords with our study depicting that the maximal odds of BP occur 1–2 years following the initiation of DPP4i. Intriguingly, the elevated odds of BP persisted even beyond 6 years after the initiation of the drug. This finding is commensurate with the study of Douros et al. [6], which attested that the risk of BP remains elevated almost 6 years after the start of the treatment. Consequently, DPP4i should be suspected as a putative trigger for BP, even if it had been started numerous years prior to the onset of BP. The delayed onset of BP following the administration of DPP4i might suggest that additional factors are required to break the tolerance to BP180. The latter is continuously maintained as might be inferred by the increased risk of BP after checkpoint inhibitors [26].

Several observational studies have attributed a more severe phenotype for DPP4i-associated BP. Ständer et al. [27] found that patients with DPP4i-associated BP had a more severe bullous component, as indicated by a higher erosion/blister BPDAI score. Correspondingly, Kridin [5] reported that patients with DPP4i-associated BP presented with significantly more extensive disease. Additionally, Patsatsi et al. [17] revealed that patients with DPP4i-associated BP had higher total BPDAI scores with a trend towards significance (41.0 vs. 34.1; *P* = 0.063). The current study was unable to measure severity scores of eligible patients but has provided indirect estimates of the disease burden and severity. Relative to other diabetic patients with BP, patients with DPP4i-associated BP had an increased frequency of dermatologic hospitalizations and a higher number of visits to outpatient dermatologists. These findings probably reflect a more severe and recalcitrant disease and provide a population-based evidence regarding an increased burden of this disease subtype.

Since CHS provides healthcare services for more than 50% of the general Israeli population, our findings feature high generalizability for the Israeli population. The retrieval of medical data from all tiers of healthcare facilities argues against selection bias and provides a comprehensive insight into the investigated question. The free access to healthcare services, negligible loss to follow-up, and inconsequential missing data all represent additional strengths of the study. The study has some limitations to acknowledge. Owing to its population-based nature, the study lacked direct immunopathological validation of the diagnosis of BP. The validity of the diagnosis, however, was substantiated by confining its documentation to certified dermatologists and dermatologic inpatient wards. In Israel, it is highly unlikely for dermatologists to base a diagnosis of BP without performing the globally acceptable immunodiagnostic essays like direct and indirect immunofluorescence [28]. Since the ethnic background of study participants was relatively homogenous, global generalizability might be lacking.

In conclusion, the current population-based study denoted that exposure to DPP4i is implicated with an 80% increase in the likelihood of subsequent BP. In an intraclass analysis, vildagliptin conferred the greatest odds of provoking BP. The risk of BP reached its peak 1–2 years following the initiation of DPP4i and remained elevated for more than 6 years following drug initiation. Patients with DPP4i-associated BP exhibited a greater disease burden. These observations imply an increased burden and corroborate previous reports about a more severe phenotype of this disease subtype. Clinicians should be aware that DPP4i might still be able to elicit BP even numerous years after drug initiation. Further studies investigating patients from different ethnic backgrounds are warranted to validate our findings.

## Supplementary Information

Below is the link to the electronic supplementary material.

Supplementary file1 (JPG 196 KB)
