# Supplementary figures and images for: Dipeptidyl-peptidase IV inhibitor (DPP4i) confers increased odds of bullous pemphigoid even years after drug initiation
Source: Arch Dermatol Res. 2022 Jan 15;315(1):33–9. doi: 10.1007/s00403-021-02317-9 (PMC9813067; doi:10.1007/s00403-021-02317-9)

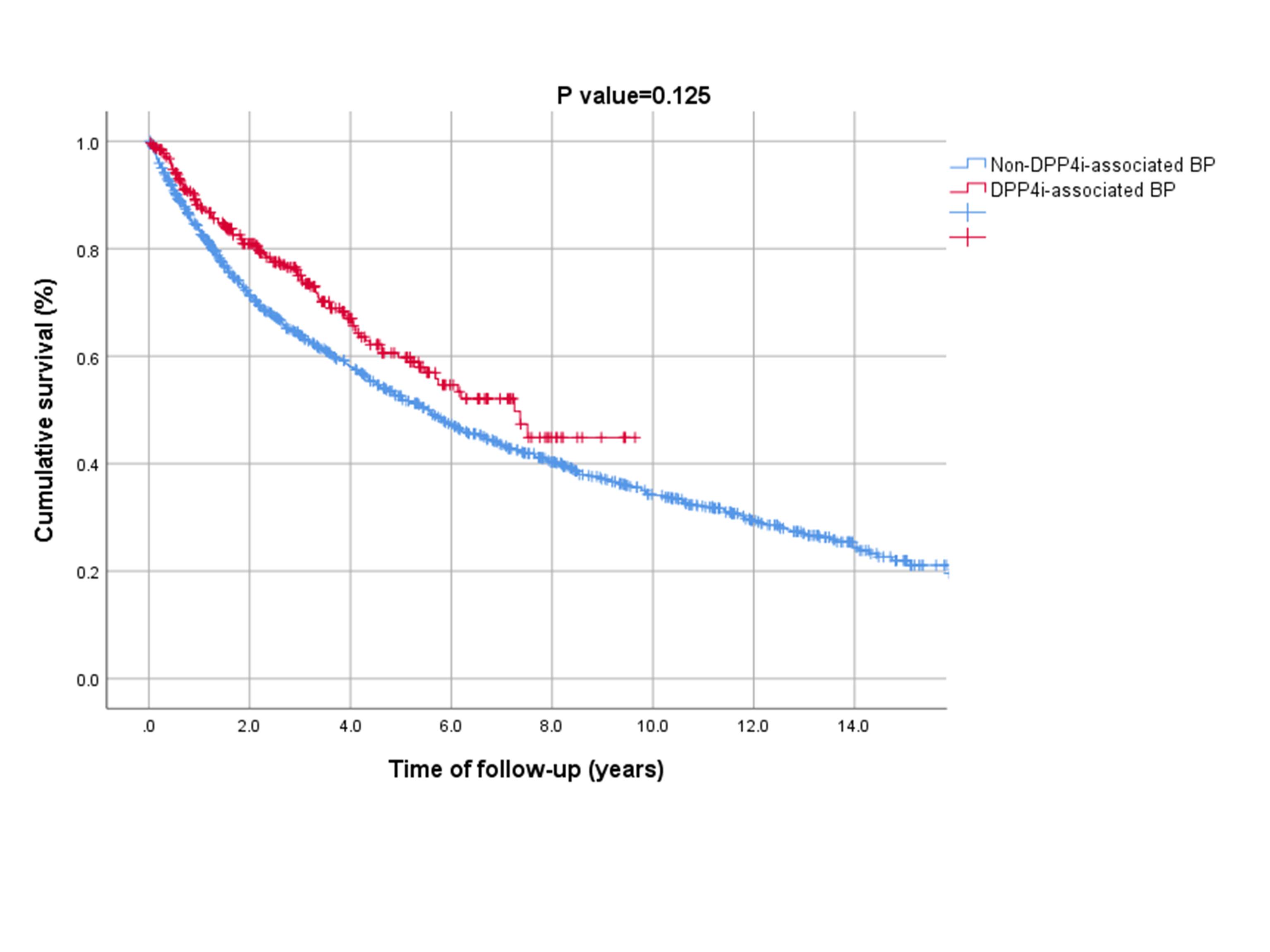

Supplement: Supplementary file 1 — Supplementary file1 (JPG 196 KB) [file 403_2021_2317_MOESM1_ESM.jpg]
